# Multi-camera real-time three-dimensional tracking of multiple flying animals

**DOI:** 10.1098/rsif.2010.0230

**Published:** 2010-07-14

**Authors:** Andrew D. Straw, Kristin Branson, Titus R. Neumann, Michael H. Dickinson

**Affiliations:** California Institute of Technology, Bioengineering, Mailcode 138-78, Pasadena, CA 91125, USA

**Keywords:** computer vision, animal behaviour, flight, manoeuvring, insects, birds

## Abstract

Automated tracking of animal movement allows analyses that would not otherwise be possible by providing great quantities of data. The additional capability of tracking in real time—with minimal latency—opens up the experimental possibility of manipulating sensory feedback, thus allowing detailed explorations of the neural basis for control of behaviour. Here, we describe a system capable of tracking the three-dimensional position and body orientation of animals such as flies and birds. The system operates with less than 40 ms latency and can track multiple animals simultaneously. To achieve these results, a multi-target tracking algorithm was developed based on the extended Kalman filter and the nearest neighbour standard filter data association algorithm. In one implementation, an 11-camera system is capable of tracking three flies simultaneously at 60 frames per second using a gigabit network of nine standard Intel Pentium 4 and Core 2 Duo computers. This manuscript presents the rationale and details of the algorithms employed and shows three implementations of the system. An experiment was performed using the tracking system to measure the effect of visual contrast on the flight speed of *Drosophila melanogaster*. At low contrasts, speed is more variable and faster on average than at high contrasts. Thus, the system is already a useful tool to study the neurobiology and behaviour of freely flying animals. If combined with other techniques, such as ‘virtual reality’-type computer graphics or genetic manipulation, the tracking system would offer a powerful new way to investigate the biology of flying animals.

## 1. Introduction

Much of what we know about the visual guidance of flight [1–4], aerial pursuit [5–9], olfactory search algorithms [10,11] and control of aerodynamic force generation [12,13] is based on experiments in which an insect was tracked during flight. To facilitate these types of studies and to enable new ones, we created a new, automated animal tracking system. A significant motivation was to create a system capable of robustly gathering large quantities of accurate data in a highly automated fashion in a flexible way. The real-time nature of the system enables experiments in which an animal's own movement is used to control the physical environment, allowing virtual-reality or other dynamic stimulus regimes to investigate the feedback-based control performed by the nervous system. Furthermore, the ability to easily collect flight trajectories facilitates data analysis and behavioural modelling using machine-learning approaches that require large amounts of data.

Our primary innovation is the use of arbitrary numbers of inexpensive cameras for markerless, real-time tracking of multiple targets. Typically, cameras with relatively high temporal resolution, such as 100 frames per second, and which are suitable for real-time image analysis (those that do not buffer their images to on-camera memory), have relatively low spatial resolution. To have high spatial resolution over a large tracking volume, many cameras are required. Therefore, the use of multiple cameras enables tracking over large, behaviourally and ecologically relevant spatial scales with high spatial and temporal resolutions while minimizing the effects of occlusion. The framework naturally allows information gathered from each camera view to incrementally improve localization. Individual views of the target thus refine the tracking estimates, even if other cameras do not see it (for example, owing to occlusions or low contrast). The use of multiple cameras also gives the system its name, *flydra*, from ‘fly’, our primary experimental animal, and the mythical Greek multi-headed serpent ‘hydra’.

Flydra is largely composed of standard algorithms, hardware and software. Our effort has been to integrate these disparate pieces of technology into one coherent, working system with the important property that the multi-target tracking algorithm operates with low latency during experiments.

### 1.1. System overview

A Bayesian framework provides a natural formalism to describe our multi-target tracking approach. In such a framework, previously held beliefs are called the *a priori*, or prior, probability distribution of the state of the system. Incoming observations are used to update the estimate of the state into the *a posteriori*, or posterior, probability distribution. This process is often likened to human reasoning, whereby a person's best guess at some value is arrived at through a process of combining previous expectations of that value with new observations that inform about the value.

The task of flydra is to find the maximum *a posteriori* (MAP) estimate of the state 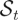 of all targets at time *t* given observations *𝒵*_1:*t*_ from all time steps (starting with the first time step to the current time step), *p*(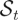
|*𝒵*_1:*t*_). Here, 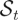 represents the state (position and velocity) of all targets, 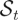 = (**s**_*t*_^1^, … **s**_*t*_^*l*_*t*_^) where *l*_*t*_ is the number of targets at time *t*. Under the first-order Markov assumption, we can factorize the posterior as1.1
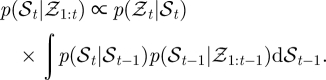


Thus, the process of estimating the posterior probability of target state at time *t* is a recursive process in which new observations are used in the model of observation likelihood *p*(𝒵_*t*_|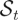). Past observations become incorporated into the prior, which combines the motion model *p*(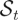
|
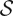
_*t*−1_) with the target probability from the previous time step *p*(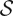
_*t*−1_|𝒵_1:*t*−1_).

Flydra uses an extended Kalman filter (EKF) to approximate the solution to equation (1.1), as described in §3.1. The observation 𝒵_*t*_ for each time step is the set of all individual low-dimensional feature vectors containing image position information arising from the camera views of the targets (§2). In fact, equation (1.1) neglects the challenges of data association (linking individual observations with specific targets) and targets entering and leaving the tracking volume. Therefore, the nearest neighbour standard filter (NNSF) data association step is used to link individual observations with target models in the model of observation likelihood (§3.2), and the state update model incorporates the ability for targets to enter and leave the tracking volume (§3.2.3). The heuristics employed to implement the system typically were optimizations with regard to real-time performance and low latency rather than a compact form, and our system only approximates the full Bayesian solution rather than perfectly implements it. Nevertheless, the remaining sections of this manuscript address their relation to the global Bayesian framework where possible. Aspects of the system which were found to be important for low-latency operation are mentioned.

**Figure 1.**
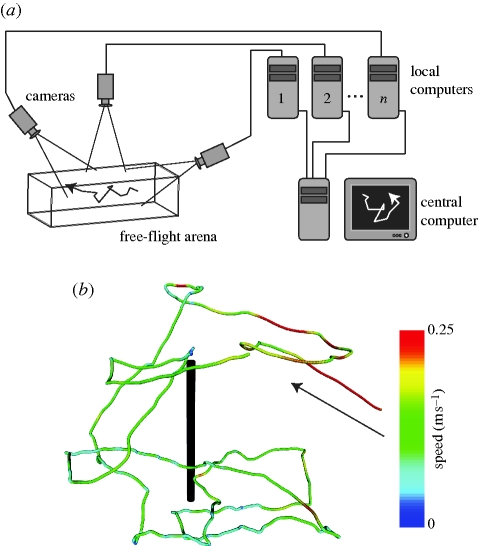
(*a*) Schematic of the multi-camera tracking system. (*b*) A trajectory of a fly (*Drosophila melanogaster*) near a dark, vertical post. Arrow indicates direction of flight at onset of tracking.

**Figure 2.**
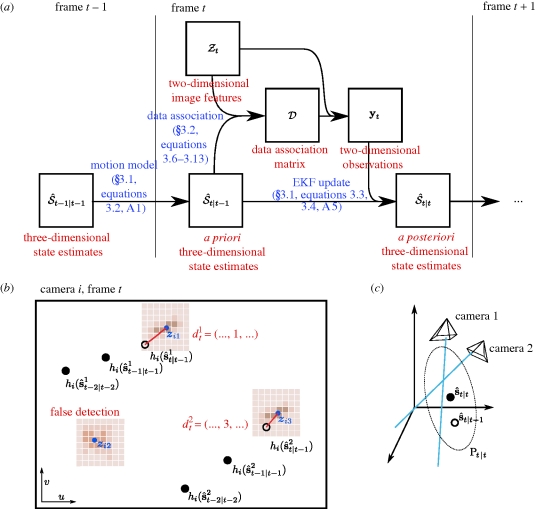
(*a*) Flowchart of operations. (*b*) Schematic of a two-dimensional camera view showing the raw images (brown), feature extraction (blue), state estimation (black), and data association (red). See §§2 and 3.2.4 for an explanation of the symbols. (*c*) Three-dimensional reconstruction using the EKF uses prior state estimates (open circle) and observations (blue lines) to construct a posterior state estimate (filled circle) and covariance ellipsoid (dotted ellipse). See appendix A for details.

**Figure 3.**
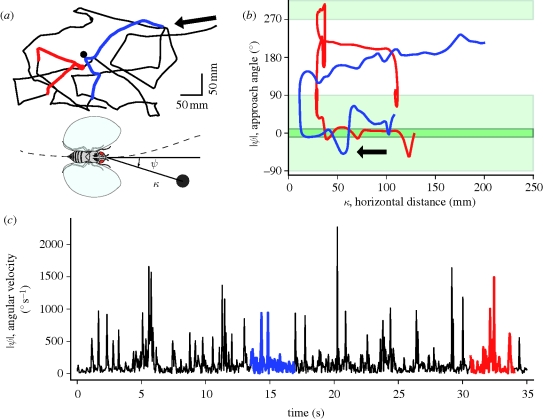
(*a*) Top view of the fly trajectory in figure 1*b*, showing several close approaches to and movements away from a dark post placed in the centre of the arena. The arrow indicates initial flight direction. Two sequences are highlighted in colour. The inset shows the coordinate system. (*b*) The time-course of attraction and repulsion to the post is characterized by flight directly towards the post until only a small distance remains, at which point the fly turns rapidly and flies away. Approach angle *ψ* is quantified as the difference between the direction of flight and bearing to the post, both measured in the horizontal plane. The arrow indicates the initial approach for the selected sequences. (*c*) Angular velocity (measured tangent to the three-dimensional flight trajectory) indicates that relatively straight flight is punctuated by saccades (brief, rapid turns).

The general form of the apparatus is illustrated in figure 1*a* and a flowchart of operations is given in figure 2*a*. Digital cameras are connected (with an IEEE 1394 FireWire bus or a dedicated gigabit ethernet cable) to image processing computers that perform a background subtraction-based algorithm to extract image features such as the two-dimensional target position and orientation in a given camera's image. From these computers, this two-dimensional information is transmitted over a gigabit ethernet LAN to a central computer, which performs two- to three-dimensional triangulation and tracking. Although the tracking results are generated and saved online, in real time as the experiment is performed, raw image sequences can also be saved for both verification purposes as well as other types of analyses. Finally, reconstructed flight trajectories, such as that of figure 1*b*, may then be subjected to further analysis (figure 3 and see figure 9).

### 1.2. Related work

Several systems have allowed manual or manually assisted digitization of the trajectories of freely flying animals. In the 1970s, Land & Collett [5] performed pioneering studies on the visual guidance of flight in blowflies and, later, in hoverflies [14,15]. By the end of the 1970s and into the 1980s, three-dimensional reconstructions using two views of flying insects were performed [6–8,16,17]. In one case, the shadow of a bee on a planar white surface was used as a second view to perform three-dimensional reconstruction [18]. Today, hand digitization is still used when complex kinematics, such as wing shape and position, are desired, such as in *Drosophila* [13], cockatoo [19] and bats [20].

Several authors have solved similar automated multi-target tracking problems using video. For example, Khan *et al.* [21] tracked multiple, interacting ants in two-dimensions from a single view using particle filtering with a Markov Chain Monte Carlo sampling step to solve the multi-target tracking problem. Later work by the same authors [22] achieved real-time speeds through the use of sparse updating techniques. Branson *et al.* [23] addressed the same problem for walking flies. Their technique uses background subtraction and clustering to detect flies in the image, and casts the data association problem as an instance of minimum weight bipartite perfect matching. In implementing flydra, we found the simpler system described here to be sufficient for tracking the position of flying flies and hummingbirds (§5). In addition to tracking in three dimensions rather than two, a key difference between the work described about and those addressed in the present work is that the interactions between our animals are relatively weak (§3.2, especially equation (3.6)), and we did not find it necessary to implement a more advanced tracker. Nevertheless, the present work could be used as the basis for a more advanced tracker, such as the one using a particle filter (e.g. [24]). In that case, the posterior from the EKF (§3.1) could be used as the proposal distribution for the particle filter. Others have decentralized the multiple object tracking problem to improve performance, especially when dealing with dynamic occlusions owing to targets occluding each other (e.g. [25,26]). Additionally, tracking of dense clouds of starlings [27–30] and fruit flies [31,32] has enabled detailed investigation of swarms, although these systems are currently incapable of operating in real time. By filming inside a corner-cube reflector, multiple (real and reflected) images allowed Bomphrey *et al.* [33] to track flies in three dimensions with only a single camera, and the tracking algorithm presented here could make use of this insight.

Completely automated three-dimensional animal tracking systems have more recently been created, such as systems with two cameras that track flies in real time [34–36]. The system of Grover *et al.* [37], similar in many respects to the one we describe here, tracks the visual hull of flies using three cameras to reconstruct a polygonal model of the three-dimensional shape of the flies. Our system, briefly described in a simpler, earlier form in Maimon *et al.* [38], differs in several ways. First, flydra has a design goal of tracking over large volumes, and, as a result of the associated limited spatial resolution (rather than owing to a lack of interest), flydra is concerned only with the location and orientation of the animal. Second, to facilitate tracking over large volumes, the flydra system uses a data association step as part of the tracking algorithm. The data association step allows flydra to deal with additional noise (false positive feature detections) when dealing with low contrast situations often present when attempting to track in large volumes. Third, our system does not attempt to maintain identity correspondence of multiple animals over extended durations, but rather stops tracking individuals when the tracking error is too high and starts tracking as new individuals when detected again. Finally, although their system operates in real time, no measurements of latency were provided by Grover *et al.* [37] with which to compare our measurements.

### 1.3. Notation

In the equations to follow, letters in a bold, roman font signify a vector, which may be specified by components enclosed in parentheses and separated by commas. Matrices are written in roman font with uppercase letters. Scalars are in italics. Vectors always act like a single column matrix, such that for vector **v** = (*a*, *b*, *c*), the multiplication with matrix 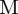 is 
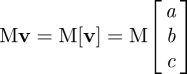
.

## 2. Two-dimensional feature extraction

The first stage of processing converts digital images into a list of feature points using an elaboration of a background subtraction algorithm. Because the image of a target is usually only a few pixels in area, an individual feature point from a given camera characterizes that camera's view of the target. In other words, neglecting missed detections or false positives, there is usually a one-to-one correspondence between targets and extracted feature points from a given camera. Nevertheless, our system is capable of successful tracking despite missing observations owing to occlusion or low contrast (§3.1) and rejecting false positive feature detections (as described in §3.2).

In the Bayesian framework, all feature points for time *t* are the observation 𝒵_*t*_. The *i*th of *n* cameras returns *m* feature points, with each point **z**_*ij*_ being a vector **z**_*ij*_ = (*u*, *v*, *α*, *β*, *θ*, *ε*) where *u* and *v* are the coordinates of the point in the image plane and the remaining components are local image statistics described below. 𝒵_*t*_ thus consists of all such feature points for a given frame 𝒵_*t*_ = {**z**_11_, … , **z**_1*m*_, … ,**z**_*n*1_, … ,**z**_*nm*_}. (In the interest of simplified notation, our indexing scheme is slightly misleading here—there may be varying numbers of features for each camera rather than always *m* as suggested.)

The process to convert a new image to a series of feature points uses a process based on background subtraction using the running Gaussian average method (reviewed in [39]). To achieve fast image processing required for real-time operation, many of these operations are performed using the high-performance *single instruction multiple data* extensions available on recent ×86 CPUs. Initially, an absolute difference image is made, where each pixel is the absolute value of the difference between the incoming frame and the background image. Feature points that exceed some threshold difference from the background image are noted and a small region around each pixel is subjected to further analysis. For the *j*th feature, the brightest point has value *β*_*j*_ in this absolute difference image. All pixels below a certain fraction (e.g. 0.3) of *β*_*j*_ are set to zero to reduce moment arms caused by spurious pixels. Feature area *α*_*j*_ is found from the 0th moment, the feature centre (*ũ*_*j*_, *ṽ*_*j*_) is calculated from the 1st moment and the feature orientation *θ*_*j*_ and eccentricity *ε*_*j*_ are calculated from higher moments. After correcting for lens distortion (§4), the feature centre is (*u*_*j*_, *v*_*j*_). Thus, the *j*th point is characterized by the vector **z**_*j*_ = (*u*_*j*_, *v*_*j*_, *α*_*j*_, *β*_*j*_, *θ*_*j*_, *ε*_*j*_). Such features are extracted on every frame from every camera, although the number of points *m* found on each frame may vary. We set the initial thresholds for detection low to minimize the number of missed detections—false positives at this stage are rejected later by the data association algorithm (§3.2).

Our system is capable of dealing with illumination conditions that vary slowly over time by using an ongoing estimate of the background luminance and its variance, which are maintained on a per-pixel basis by updating the current estimates with data from every 500th frame (or other arbitrary interval). A more sophisticated two-dimensional feature extraction algorithm could be used, but we have found this scheme to be sufficient for our purposes and sufficiently simple to operate with minimal latency.

While the real-time operation of flydra is essential for experiments modifying sensory feedback, another advantage of an online tracking system is that the amount of data required to be saved for later analysis is greatly reduced. By performing only two-dimensional feature extraction in real time, to reconstruct three-dimensional trajectories later, only the vectors **z**_*j*_ need be saved, resulting in orders of magnitude less data than the full-frame camera images. Thus, to achieve solely the low data rates of real-time tracking, the following sections dealing with three dimensions are not necessary to be implemented for this benefit of real-time use. Furthermore, raw images taken from the neighbourhood of the feature points could also be extracted and saved for later analysis, saving slightly more data, but still at rates substantially less than the full camera frames provide. This fact is particularly useful for cameras with a higher data rate than hard drives can save, and such a feature is implemented in flydra.

**Figure 4.**
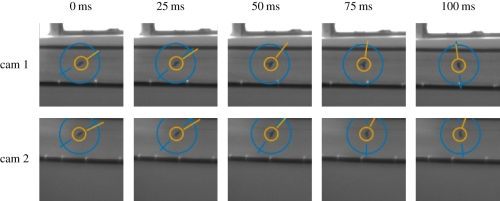
Raw images, two-dimensional data extracted from the images, and overlaid computed three-dimensional position and body orientation of a hummingbird (*Calypte anna*). In these images, a blue circle is drawn centred on the two-dimensional image coordinates (*ũ*, *ṽ*). The blue line segment is drawn through the detected body axis (*θ* in §3.2) when eccentricity (*ɛ*) of the detected object exceeds a threshold. The orange circle is drawn centred on the three-dimensional estimate of position (*x*, *y*, *z*) reprojected through the camera calibration matrix P, and the orange line segment is drawn in the direction of the three-dimensional body orientation vector.

Figure 4 shows the parameters (*u*, *v*, *θ*) from the two-dimensional feature extraction algorithm during a hummingbird flight. These two-dimensional features, in addition to three-dimensional reconstructions, are overlaid on raw images extracted and saved using the real-time image extraction technique described above.

## 3. Multi-target tracking

The goal of flydra, as described in §1.1, is to find the MAP estimate of the state of all targets. For simplicity, we model interaction between targets in a very limited way. Although in many cases the animals we are interested in tracking do interact (for example, hummingbirds engage in competition in which they threaten or even contact each other), mathematically limiting the interaction facilitates a reduction in computational complexity. First, the process update is independent for each *k*th animal3.1
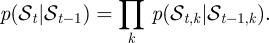


Second, we implemented only a slight coupling between targets in the data association algorithm. Thus, the observation likelihood model *p*(𝒵_*t*_|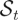) is independent for each target with the exception described in §3.2.1, and making this assumption allows use of the NNSF as described below.

Modelling individual target states as mostly independent allows the problem of estimating the MAP of joint target state 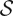 to be treated nearly as *l* independent, smaller problems. One benefit of making the assumption of target independence is that the target tracking and data association parts of our system are parallelizable. Although not yet implemented in parallel, our system is theoretically capable of tracking very many (tens or hundreds) targets simultaneously with low latency on a computer with sufficiently many processing units.

The cost of this near-independence assumption is reduced tracking accuracy during periods of near contact (§3.2.1). Data from these periods could be analysed later using more sophisticated multi-target tracking data association techniques, presumably in an offline setting, especially because such periods could be easily identified using a simple algorithm. All data presented in this paper used the procedure described here.

### 3.1. Kalman filtering

The standard EKF approximately estimates statistics of the posterior distribution (equation (1.1)) for nonlinear processes with additive Gaussian noise (details are given in appendix A). To use this framework, we make the assumption that noise in the relevant processes is Gaussian. Additionally, our target independence assumption allows a single Kalman filter implementation to be used for each tracked target. The EKF estimates state and its covariance based on a prior state estimate and incoming observations by using models of the state update process, the observation process, and estimates of the noise of each process.

We use a linear model for the dynamics of the system and a nonlinear model of the observation process. Specifically, the time evolution of the system is modelled with the linear discrete stochastic model3.2
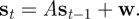


We treat the target as an orientation-free particle, with state vector **s** = (*x*, *y*, *z*, *ẋ*, *ẏ*, *ż*) describing position and velocity in three-dimensional space. The process update model A represents, in our case, the laws of motion for a constant velocity particle


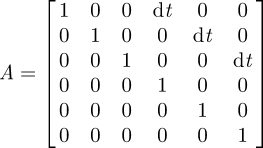


with d*t* being the time step. Manoeuvring of the target (deviation from the constant velocity) is modelled as noise in this formulation. The random variable **w** represents this process update noise with a normal probability distribution with zero mean and the process covariance matrix Q. Despite the use of a constant velocity model, the more complex trajectory of a fly or other target is accurately estimated by updating the state estimate with frequent observations.

**Figure 5.**
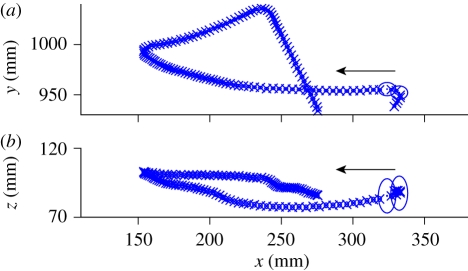
Two seconds of a reconstructed *Drosophila* trajectory. (*a*) Top view. (*b*) Side view of same trajectory. Kalman filter-based estimates of fly position **s** are plotted as dots at the centre of ellipsoids, which are the projections of the multi-variate normal specified by the covariance matrix P. Additionally, position estimated directly by triangulation of two-dimensional point locations (see appendix B) is plotted with crosses. The fly began on the right and flew in the direction denoted by the arrow. Note that for two frames near the beginning, only a single camera contributed to the tracking and the error estimate increased.

For a given data association hypothesis (§3.2), a set of observations is available for target *k*. A nonlinear observation model, requiring use of an EKF, is used to describe the action of a projective camera (equations (3.3) and (3.4)). This allows observation error to be modelled as Gaussian noise on the image plane. Furthermore, during tracking, triangulation happens only implicitly, and error estimates of target position are larger along the direction of the ray between the target and the camera centre. (To be clear, explicit triangulation *is* performed during the initialization of a Kalman model target, as explained in §3.2.) Thus, observations from alternating single cameras on successive frames would be sufficient to produce a three-dimensional estimate of target position. For example, figure 5 shows a reconstructed fly trajectory in which two frames were lacking data from all but one camera. During these two frames, the estimated error increased, particularly along the camera-fly axis, and no triangulation was possible. Nevertheless, an estimate of three-dimensional position was made and appears reasonable. The observation vector **y** = (*u*_1_, *v*_1_, *u*_2_, *v*_2_, … , *u*_*n*_, *v*_*n*_) is the vector of the distortion-corrected image points from *n* cameras. The nonlinear observation model relates **y**_*t*_ to the state **s**_*t*_ by3.3
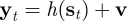
where **s**_*t*_ is the state at time *t*, the function *h* models the action of the cameras and **v** is observation noise. The vector-valued *h*(**s**) is the concatenation of the image points found by applying the image point equations (equations (B 2) and (B 3) in appendix B.) to each of the *n* cameras3.4
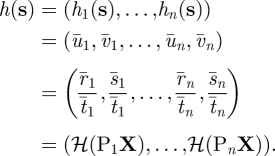


The overbar (-) denotes a noise-free prediction to which the zero-mean noise vector **v** is added, and **X** is the homogeneous form of the first three components of **s**. The random variable **v** models the observation noise as normal in the image plane with zero mean and covariance matrix R.

At each time step *t*, the EKF formulation is then used to estimate the state ŝ in addition to the error P (see appendix A). Together, the data associated with each target is *Γ* = {ŝ, P}. With the possibility of multiple targets being tracked simultaneously, the *k*th target is assigned *Γ*^*k*^.

One issue we faced when implementing the Kalman filter was parameter selection. Our choice of parameters was done through educated guesses followed by an iterated trial-and-error procedure using several different trajectories' observations. The parameters that resulted in trajectories closest to those seen by eye and with least magnitude error estimate P were used. We obtained good results, for fruit flies measured under the conditions of our set-ups, with the process covariance matrix Q being diagonal, with the first three entries being 100 mm^2^ and the next three being 0.25 (m^2^ s^−2^). Therefore, our model treats manoeuvring as position and velocity noise. For the observation covariance matrix *R*, we found good results with a diagonal matrix with entries of 1, corresponding to variance of the observed image positions of one pixel. Parameter selection could be automated by an expectation–maximization algorithm, but we found this was not necessary.

Another issue is missing data—in some time steps, all views of the fly may be occluded or low contrast, leaving a missing value of **y** for that time step. In those cases, we simply set the *a posteriori* estimate to the *a priori* prediction, as follows from equation (1.1). In these circumstances, the error estimate P grows by the process covariance Q, and does not get reduced by (non-existent) new observations. This follows directly from the Kalman filter equations (appendix A). If too many successive frames with no observations occur, the error estimate will exceed a threshold and tracking will be terminated for that target (described in §3.2.3).

### 3.2. Data association

One simplification made in the system overview (§1.1) was to neglect the data association problem—the assignment of observations to targets. We address the problem by marginalizing the observation likelihood across hidden data association variables 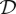, where each 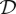 corresponds to a different hypothesis about how the feature points correspond with the targets. Thus, the model of observation likelihood from equation (1.1) becomes3.5
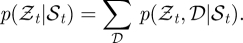


In fact, computing probabilities across all possible data association hypotheses 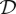 across multiple time steps would result in a combinatorial explosion of possibilities. Among the various means of limiting the amount of computation required by limiting the number of hypotheses considered, we have chosen a simple method, the NNSF data association algorithm run on each target independently [40]. This algorithm is sufficiently efficient to operate in real time for typical conditions of our system. Thus, we approximate the sum of all data association hypotheses with the single best hypothesis 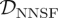, defined to be the NNSF output for each of the *k* independent targets3.6
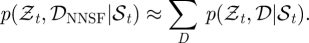


This implies that we assume hypotheses other than 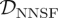 have vanishingly small probability. Errors owing to this assumption being false could be corrected in a later, offline pass through the data keeping track of more data association hypotheses using other algorithms.


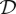
 is a matrix with each column being the data association vector for target *k* such that 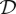 = [***d***^1^ … ***d***^*k*^ … ]. This matrix has *n* rows (the number of cameras) and *l* columns (the number of active targets). The data association vector ***d***^*k*^ for target *k* has *n* elements of value null or index *j* of the feature **z**_*j*_ assigned to that target. As described below (§3.2.4), these values are computed from the predicted location of the target and the features returned from the cameras.

#### 3.2.1. Preventing track merging

One well-known potential problem with multi-target tracking is the undesired merging of target trajectories if targets begin to share the same observations. Before implementing the following rule, flydra would sometimes suffer from this merging problem when tracking hummingbirds engaged in territorial competition. In such fights, male hummingbirds often fly directly at each other and come in physical contact. To prevent the two trajectories from merging in such cases, a single pass is made through the data association assignments after each frame. In the case that more than one target was assigned the exact same subset of feature points, a comparison is made between the observation and the predicted observation. In this case, only the target corresponding to the closest prediction is assigned the data, and the other target is updated without any observation. We found this procedure to require minimal additional computational cost, while still being effective in preventing trajectory merging.

#### 3.2.2. NNSF and generative model of image features

To implement the NNSF algorithm, we implement a generative model of feature appearance based on the prior estimate of target state. By predicting target position in an incoming frame based on prior information, the system selects two-dimensional image points as being likely to come from the target by gating unlikely observations, thus limiting the amount of computation performed.

**Figure 6.**
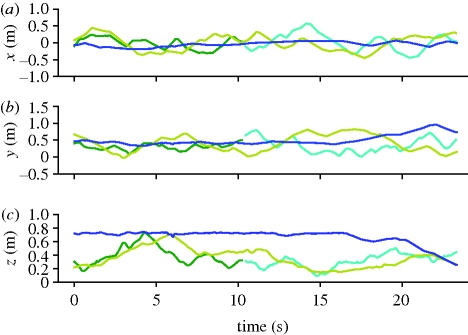
Multiple flies tracked simultaneously. Each automatically segmented trajectory is plotted in its own colour. Note that the dark green and cyan trajectories probably came from the same fly which, for a period near the 10th second, was not tracked owing to a series of missed detections or leaving the tracking volume (§3.2). (*a*) First horizontal axis (*x*). (*b*) Second horizontal axis (*y*). (*c*) Vertical axis (*z*).

Recall from §2 that for each time *t* and camera *i*, *m* feature points are found with the *j*th point being **z**_*j*_ = (*u*_*j*_, *v*_*j*_, *α*_*j*_, *β*_*j*_, *θ*_*j*_, *ɛ*_*j*_). The distortion-corrected image coordinates are (*u*, *v*), while *α* is the area of the object on the image plane measured by thresholding of the difference image between the current and background image, *β* is an estimate of the maximum difference within the difference image, and *θ* and *ɛ* are the slope and eccentricity of the image feature. Each camera may return multiple candidate points per time step, with all points from the *i*th camera represented as Z_*i*_, a matrix whose columns are the individual vectors **z**_*j*_, such that Z_*i*_ = [**z**_1_ … **z**_*m*_]. The purpose of the data association algorithm is to assign each incoming point **z** to an existing Kalman model *Γ*, to initialize a new Kalman model, or attribute it to a false positive (a null target). Furthermore, old Kalman models for which no recent observations exist owing to the target leaving the tracking volume must be deleted. The use of such a data association algorithm allows flydra to track multiple targets simultaneously, as in figure 6, and, by reducing computational costs, allows the two-dimensional feature extraction algorithm to return many points to minimize the number of missed detections.

#### 3.2.3. Entry and exit of targets

How does our system deal with existing targets losing visibility owing to leaving the tracking volume, occlusion or lowered visual contrast? What happens when new targets become visible? We treat such occurrences as part of the update model in the Bayesian framework of §1.1. Thus, in the terminology from that section, our motion model for all targets *p*(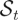
|
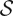
_*t*−1_) includes the possibility of initializing a new target or removing an existing target. This section describes the procedure followed.

For all data points **z** that remained ‘unclaimed’ by the predicted locations of pre-existing targets (see §3.2.4 below), we use an unguided hypothesis testing algorithm. This triangulates a hypothesized three-dimensional point for every possible combination of 2, 3, … , *n* cameras, for a total of 
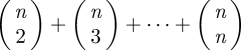
 combinations. Any three-dimensional point with reprojection error less than an arbitrary threshold using the greatest number of cameras is then used to initialize a new Kalman filter instance. The initial state estimate is set to that three-dimensional position with zero velocity and a relatively high error estimate. Tracking is stopped (a target is removed) once the estimated error P exceeds a threshold. This most commonly happens, for example, when the target leaves the tracking area and thus receives no observations for a given number of frames.

#### 3.2.4. Using incoming data

Ultimately, the purpose of the data association step is to determine which image feature points will be used by which target. Given the target independence assumption, each target uses incoming data independently. This section outlines how the data association algorithm is used to determine the feature points treated as the observation for a given target.

To use the Kalman filter described in §3.1, observation vectors must be formed from the incoming data. False positives must be separated from correct detections, and, because multiple targets may be tracked simultaneously, correct detections must be associated with a particular Kalman model. At the beginning of processing for each time step *t* for the *k*th Kalman model, a prior estimate of target position and error *Γ*_*t*|*t*−1_^*k*^ = {**ŝ**_*t*|*t*−1_^*k*^, P_*t*|*t*−1_^*k*^} is available. It must be determined which, if any, of the *m* image points from the *i*th camera is associated with the *k*th target. Due to the real-time requirements for our system, flydra gates incoming detections on simple criteria before performing more computationally intensive tasks.

For target *k* at time *t*, the data association function *g* is3.7
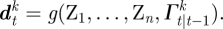
This is a function of the image points Z_*i*_ from each of the *n* cameras and the prior information for target *k*. The assignment vector for the *k*th target, ***d***^*k*^, defines which points from which cameras view a target. This vector has a component for each of the *n* cameras, which is either null (if that camera does not contribute) or is the column index of Z_*i*_ corresponding to the associated point. Thus, ***d***^*k*^ has length *n*, the number of cameras, and no camera may view the same target more than once. Note, the *k* and *t* superscript and subscript on ***d*** indicate the assignment vector is for target *k* at time step *t*, whereas below (equation (3.8)), the subscript *i* is used to indicate the *i*th component of the vector ***d***.

The data association function *g* may be written in terms of the components of ***d***. The *i*th component is the index of the columns of Z_*i*_ that maximizes likelihood of the observation given the predicted target state and error and is defined to be3.8
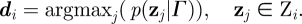


Our likelihood function gates detections based on two conditions. First, the incoming detected location (*u*_*j*_, *v*_*j*_) must be within a threshold Euclidean distance from the estimated target location projected on the image. The Euclidean distance on the image plane is3.9
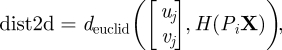
where ℋ(P_*i*_**X**) finds the projected image coordinates of **X**, where **X** is the homogeneous form of the first three components of **s**, the expected three-dimensional position of the target. The function ℋ and camera matrix P_*i*_ are described in appendix B. The gating can be expressed as an indicator function3.10
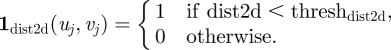


Second, the area of the detected object (*α*_*j*_) must be greater than a threshold value, expressed as3.11
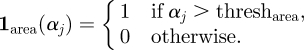


If these conditions are met, the distance of the ray connecting the camera centre and two-dimensional point on the image plane (*u*_*j*_, *v*_*j*_) from the expected three-dimensional location **ā** is used to further determine likelihood. We use the Mahalanobis distance, which for a vector **a** with an expected value of **ā** with covariance matrix *Σ* is3.12
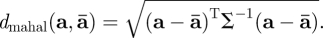


Because the distance function is convex for a given **ā** and *Σ*, we can solve directly for the closest point on the ray, by setting **ā** equal to the first three terms of **ŝ**_*t*|*t*−1_ and *Σ* to the upper left 3 × 3 submatrix of P_*t*|*t*−1_. Then, if the ray is a parametrized line of the form ℒ(*s*) = *s* · (*a*, *b*, *c*) + (*x*, *y*, *z*) where (*a*, *b*, *c*) is the direction of the ray formed by the image point (*u*, *v*) and the camera centre and (*x*, *y*, *z*) is a point on the ray, we can find the value of *s* for which the distance between ℒ(*s*) and **ā** is minimized by finding the value of *s* where derivative of *d*_mahal_ (ℒ(*s*), **ā**) is zero. If we call this closest point **a** and combine equations (3.10)–(3.12), then our likelihood function is3.13
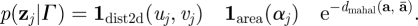


Note that, owing to the multiplication, if either of the first two factors is zero, the third (and more computationally expensive) condition need not be evaluated.

## 4. Camera and lens calibration

Camera calibrations may be obtained in any way that produces camera calibration matrices (described in appendix B) and, optionally, parameters for a model of the nonlinear distortions of cameras. Good calibrations are critical for flydra because, as target visibility changes from one subset of cameras to another subset, any misalignment of the calibrations will introduce artefactual movement in the reconstructed trajectories. Typically, we obtain camera calibrations in a two-step process. First, the direct linear transformation (DLT) algorithm [41] directly estimates camera calibration matrices P_*i*_ that could be used for triangulation as described in appendix B. However, because we use only about 10 manually digitized corresponding two-/three-dimensional pairs per camera, this calibration is of relatively low precision and, as performed, ignores optical distortions causing deviations from the linear simple pinhole camera model. Therefore, an automated *Multi-Camera Self Calibration Toolbox* [42] is used as a second step. This toolbox uses inherent numerical redundancy when multiple cameras are viewing a common set of three-dimensional points through use of a factorization algorithm [43] followed by bundle adjustment (reviewed in §18.1 of [44]). By moving a small LED point light source through the tracking volume (or, indeed, a freely flying fly), hundreds of corresponding two-/three-dimensional points are generated which lead to a single, overdetermined solution which, without knowing the three-dimensional positions of the LED, is accurate up to a scale, rotation and translation. The camera centres, either from the DLT algorithm or measured directly, are then used to find the best scale, rotation and translation. As part of the *Multi-Camera Self Calibration Toolbox*, this process may be iterated with an additional step to estimate nonlinear camera parameters such as radial distortion [42] using the *Camera Calibration Toolbox* of Bouguet [45]. Alternatively, we have also implemented the method of Prescott & McLean [46] to estimate radial distortion parameters before use of Svoboda's toolbox, which we found necessary when using wide angle lenses with significant radial distortion (e.g. 150 pixels in some cases).

## 5. Implementation and evaluation

We built three different flydra systems: a five camera, six computer 100 fps system for tracking fruit flies in a 0.3 × 0.3 × 1.5 m arena (e.g. figures 1 and 9; [38], which used the same hardware but a simpler version of the tracking software), an 11-camera, nine-computer 60 fps system for tracking fruit flies in a large—2 m diameter × 0.8 m high—cylinder (e.g. figure 5) and a four camera, five computer 200 fps system for tracking hummingbirds in a 1.5 × 1.5 × 3 m arena (e.g. figure 4). Apart from the low-level camera drivers, the same software is running on each of these systems.

We used the Python computer language to implement flydra. In particular, the open source Motmot camera software serves as the basis for the image acquisition and real-time image processing [47]. The motmot.libcamiface documentation contains information about what cameras are compatible, and the motmot.realtime_image_analysis package contains the algorithms for the two-dimensional real-time image processing. Several other pieces of software, most of which are open source, are instrumental to this system: PyTables, numpy, scipy, Pyro, wxPython, tvtk, VTK, matplotlib, PyOpenGL, pyglet, Pyrex, cython, ctypes, Intel IPP, OpenCV, ATLAS, libdc1394, PTPd, gcc and Ubuntu. We used Intel Pentium 4 and Core 2 Duo-based computers.

The quality of three-dimensional reconstructions was verified in two ways. First, the distance between two-dimensional points projected from a three-dimensional estimate derived from the originally extracted two-dimensional points is a measure of calibration precision. For all figures shown, the mean reprojection error was less than one pixel, and for most cameras in most figures, was less than 0.5 pixels. Second, to determine accuracy, we verified the three-dimensional coordinates and distances between coordinates measured through triangulation against values measured physically. For two such measurements in the system shown in figure 1, these values were within 4 per cent. So, in general, the system appears to have high precision (low reprojection error), but slighly worse accuracy. Because we are using standard calibration and estimation algorithms, we did not perform a more detailed groundtruth analysis of position estimates.

**Figure 7.**
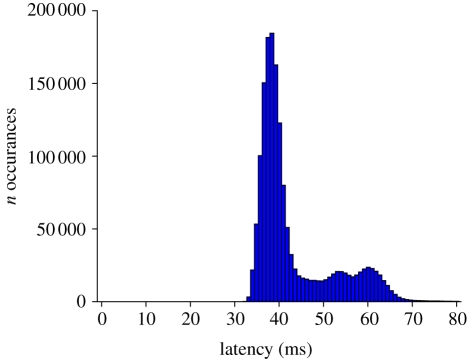
Latency of one tracking system. A histogram of the latency of three-dimensional reconstruction was generated after tracking 20 flies for 18 h. Median latency was 39 ms.

We measured the latency of the three-dimensional reconstruction by synchronizing several clocks involved in our experimental setup and then measuring the duration between onset of image acquisition and completion of the computation of target position. When flydra is running, the clocks of the various computers are synchronized to within 1 ms by PTPd, the precise time protocol daemon, an implementation of the IEEE 1588 clock synchronization protocol [48]. Additionally, a microcontroller (AT90USBKEY, Atmel, USA) running custom firmware is connected over USB to the central reconstruction computer and is synchronized using an algorithm similar to PTPd, allowing the precise time of frame trigger events to be known by processes running within the computers. Measurements were made of the latency between the time of the hardware trigger pulse generated on the microcontroller to start acquisition of frame *t* and the moment the state vector **ŝ**_*t*|*t*_ was computed. These measurements were made with a central computer being a 3 GHz Intel Core 2 Duo CPU. As shown in figure 7, the median three-dimensional reconstruction timestamp is 39 ms. Further investigation showed the make-up of this delay. From the specifications of the cameras and bus used, 19.5 ms is a lower bound on the latency of transferring the image across the IEEE 1394 bus (and could presumably be reduced by using alternative technologies such as Gigabit Ethernet or Camera Link). Further measurements on this system showed that two-dimensional feature extraction takes 6–12 ms, and that image acquisition and two-dimensional feature extraction together take 26–32 ms. The remainder of the latency to three-dimensional estimation is owing to network transmission, triangulation and tracking, and, most likely, non-optimal queueing and ordering of data while passing it between these stages. Further investigation and optimization have not been performed. Although we have not calculated latency in a similar way in the 200 fps hummingbird tracking GigE system, the computers are significantly faster and therefore two-dimensional feature extraction takes less than 5 ms on Core 2 Duo computers. Because the GigE bus is approximately twice as fast as the 1394, the similarly sized images arrive with half the latency. We therefore expect median latency in this system to be about 25 ms.

## 6. Experimental possibilities

A few examples serve to illustrate some of the capabilities of flydra. We are actively engaged in understanding the sensory-motor control of flight in the fruit fly *Drosophila melanogaster*. Many basic features of the effects of visual stimulation on the flight of flies are known, and the present system allows us to characterize these phenomena in substantial detail. For example, the presence of a vertical landmark such as a black post on a white background greatly influences the structure of flight, causing flies to ‘fixate’, or turn towards, the post [49]. By studying such behaviour in free flight (e.g. figure 3), we have found that flies approach the post until some small distance is reached, and then often turn rapidly and fly away.

**Figure 8.**
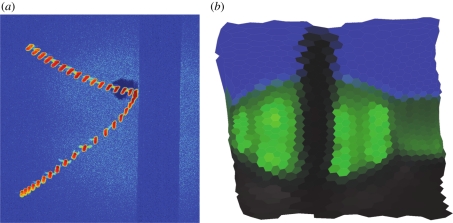
Example applications for flydra. (*a*) By tracking flies in real time, we can trigger high speed video cameras (6000 fps) to record interesting events for later analysis. (*b*) A simulated reconstruction of the visual scene viewed through the left eye of *Drosophila*. Such visual reconstructions can be used to simulate neural activity in the visual system [50,51]. In this simulated view, a three-dimensional model of the experimental environment, with green backlighting and a black vertical post, was used in conjunction with an optical model of a fly eye and the fly's pose estimate to render an image approximating that seen by a fly during an experiment.

Because we have an online estimate of fly velocity and position in ŝ, we can predict the future location of the fly. Of course, the quality of the prediction declines with the duration of extrapolation, but it is sufficient for many tasks, even with the latency taken by the three-dimensional reconstruction itself. One example is the triggering of high resolution, high speed cameras (e.g. 1024 × 1024 pixels, 6000 frames per second as shown in figure 8*a*). Such cameras typically buffer their images to RAM and are downloaded offline. We can construct a trigger condition based on the position of an animal (or a recent history of position, allowing triggering only on specific manoeuvres). Figure 8*a* shows a contrast-enhancing false colour montage of a fly making a close approach to a post before leaving. By studying precise movements of the wings and body in addition to larger-scale movement through the environment, we are working to understand the neural and biomechanical mechanisms underlying control of flight.

An additional possibility enabled by low latency three-dimensional state estimates is that visual stimuli can be modulated to produce a ‘virtual reality’ environment in which the properties of the visual feedback loop can be artificially manipulated [35,36,52,53]. In these types of experiments, it is critical that moving visual stimuli do not affect the tracking. For this reason, we illuminate flies with near-IR light and use high pass filters in front of the camera lenses (e.g. R-72, Hoya Corporation). Visual cues provided to the flies are in the blue-green range.

By estimating the orientation of the animal, approximate reconstructions of the visual stimulus experienced by the animal may be made. For example, to make figure 8*b*, a simulated view through the compound eye of a fly, we assumed that the fly head, and thus eyes, was oriented tangent to the direction of travel, and that the roll angle was fixed at zero. This information, together with a three-dimensional model of the environment, was used to generate the reconstruction [50,51]. Such reconstructions are informative for understanding the nature of the challenge of navigating visually with limited spatial resolution. Although it is known that flies do move their head relative to their longitudinal body axis, these movements are generally small [2], and thus the errors resulting from the assumptions listed above could be reduced by estimating body orientation using the method described in appendix B. Because a fly's eyes are fixed to its head, further reconstruction accuracy could be gained by fixing the head relative to the body (by gluing the head to the thorax), although the behavioural effects of such a manipulation would need to be investigated. Nevertheless, owing to the substantial uncertainties involved in estimating the pose of an insect head, investigations based on such reconstructions would need to be carefully validated.

Finally, because of the configurability of the system, it is feasible to consider large-scale tracking in naturalistic environments that would lead to greater understanding of the natural history of flies [54] or other animals.

## 7. Effect of contrast on speed regulation in *Drosophila*

At low levels of luminance contrast, when differences in the luminance of different parts of a scene are minimal, it is difficult or impossible to detect motion of visual features. In flies, which judge self-motion (in part) using visual motion detection, the exact nature of contrast sensitivity has been used as a tool to investigate the fundamental mechanism of motion detection using electrophysiological recordings. At low contrast levels, these studies have found a quadratic relationship between membrane potential and luminance contrast [55–57]. This result is consistent with Hassenstein–Reichardt correlator model for elementary motion detection (the HR-EMD, [58]), and these findings are part of the evidence to support the hypothesis that the fly visual system implements something very similar to this mathematical model.

Despite these and other electrophysiological findings suggesting the HR-EMD may underlie fly motion sensitivity, studies of flight speed regulation and other visual behaviours in freely flying flies [59] and honey bees [60–62] show that the free-flight behaviour of these insects is inconsistent with a flight velocity regulator based on a simple HR-EMD model. More recently, Baird *et al.* [63] have shown that over a large range of contrasts, flight velocity in honey bees is nearly unaffected by contrast. As noted by those authors, however, their set-up was unable to achieve true zero contrast owing to imperfections with their apparatus. They suggest that contrast adaptation [64] may have been responsible for boosting the responses to low contrasts and attenuating responses to high contrast. This possibility was supported by the finding that forward velocity was better regulated at the nominal ‘zero contrast’ condition than in the presence of an axial stripe, which may have had the effect of preventing contrast adaptation while provide no contrast perpendicular to the direction of flight [63].

**Figure 9.**
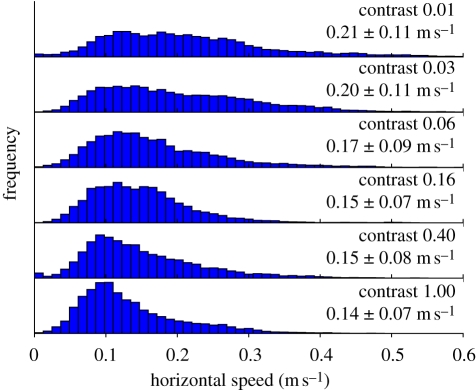
*Drosophila melanogaster* maintain a lower flight speed with lower variability as visual contrast is increased. Mean and standard deviation of flight speeds are shown in text, and each histogram is normalized to have equal area. Ambient illumination reflected from one surface after being scattered from other illuminated surfaces slightly reduced contrast from the nominal values shown here.

We tested the effect of contrast on the regulation of flight speed in *Drosophila melanogaster*, and the results are shown in figure 9. In this set-up, we found that when contrast was sufficiently high (Michelson contrast ≥ 1.6), flies regulated their speed to a mean speed of 0.15 m s^−1^ with a standard deviation of 0.07. As contrast was lowered, the mean speed increased, as did variability of speed, suggesting that speed regulation suffered owing to a loss of visual feedback. To perform these experiments, a computer projector (DepthQ, modified to remove colour filter wheel, Lightspeed Design, USA) illuminated the long walls and floor of a 0.3 × 0.3 × 1.5 m arena with a regular checkerboard pattern (5 cm^2^) of varying contrast and fixed luminance (2 cd m^−2^). The test contrasts were cycled, with each contrast displayed for 5 min. Twenty females flies were released into the arena and tracked over 12 h. Any flight segments more than 5 cm from the walls, floor or ceiling were analysed, although for the majority of the time flies were standing or walking on the floors or walls of the arena. Horizontal flight speed was measured as the first derivative of position in the XY direction, and histograms were computed with each frame constituting a single sample. Because the identity of the flies could not be tracked for the duration of the experiment, the data contain pseudo-replication—some flies probably contributed more to the overall histogram than others. Nevertheless, the results from three separate experimental days with 20 new flies tested on each day were each qualitatively similar to the pooled results shown, which include 1760 total seconds of flight in which tracked fly was 5 cm or greater from the nearest arena surface and were acquired during 30 cumulative hours.

The primary difference between our findings on the effect of contrast on flight speed in *Drosophila melanogaster* compared with that found in honey bees by [63] is that at low contrasts (below 0.16 Michelson contrast), flight speed in *Drosophila* is faster and more variable. This difference could be owing to several non-mutually exclusive factors: (i) our arena may have fewer imperfections which create visual contrast; (ii) fruit flies may have a lower absolute contrast sensitivity than honey bees; (iii) fruit flies may have a lower contrast sensitivity at the luminance level of the experiments; (iv) fruit flies may have less contrast adaptation ability; or (v) fruit flies may employ an alternate motion detection mechanism.

Despite the difference between the present results in fruit flies from those of honey bees at low contrast levels, at high contrasts (above 0.16 Michelson contrast for *Drosophila*) flight speed in both species was regulated around a constant value. This suggests that the visual system has little trouble estimating self-motion at these contrast values and that insects regulate flight speed about a set point using visual information.

## 8. Conclusions

The highly automated and real-time capabilities of our system allow unprecedented experimental opportunities. We are currently investigating the object approach and avoidance phenomenon of fruit flies illustrated in figure 3. We are also studying manoeuvring in solitary and competing hummingbirds and the role of manoeuvring in establishing dominance. One of the opportunities made possible by the molecular biological revolution are powerful new tools that can be used to visualize and modify the activity of neurons and neural circuits. By precisely quantifying high level behaviours, such as the object attraction/repulsion described above, we hope to make use of these tools to approach the question of how neurons contribute to the process of behaviour.

## Appendix A. Extended Kalman filter

The EKF was used as described in §3.1. Here, we give the equations using the notation of this paper. Note that because only the observation process is nonlinear, the process dynamics are specified with (linear) matrix A.

The *a priori* predictions of these values based on the previous frame's posterior estimates are

A 1

and

A 2

To incorporate observations, a gain term *K* is calculated to weight the innovation arising from the difference between the *a priori* state estimate **ŝ**_*t*|*t*−1_ and the observation ***y***

A 3

The observation matrix, H_*t*_, is defined to be the Jacobian of the observation function (equation (3.4)) evaluated at the expected state

A 4

The posterior estimates are then

A 5

and

A 6

## Appendix B. Triangulation

The basic two- to three-dimensional calculation finds the best three-dimensional location for two or more two-dimensional camera views of a point, and is implemented using a linear least-squares fit of the intersection of *n* rays defined by the two-dimensional image points and three-dimensional camera centres of each of the *n* cameras [44]. After correction for radial distortion, the image of a three-dimensional point on the *i*th camera is (*u*_*i*_, *v*_*i*_). For mathematical reasons, it is convenient to represent this two-dimensional image point in homogeneous coordinates

B 1

such that *u*_*i*_ = *r*_*i*_/*t*_*i*_ and *v*_*i*_ = *s*_*i*_/*t*_*i*_. For convenience, we define the function ℋ to convert from homogeneous to Cartesian coordinates, thus

B 2

The 3 × 4 camera calibration matrix P_*i*_ models the projection from a three-dimensional homogeneous point **X** = (*X*_1_, *X*_2_, *X*_3_, *X*_4_) (representing the three-dimensional point with inhomogeneous coordinates (*x*, *y*, *z*) = (*X*_1_/*X*_4_, *X*_2_/*X*_4_, *X*_3_/*X*_4_)) into the image point:

B 3

By combining the image point equation (B 3) from two or more cameras, we can solve for **X** using the homogeneous linear triangulation method based on the singular value decomposition as described in [44], §§12.2 and A5.3).

A similar approach can be used for reconstructing the orientation of the longitudinal axis of an insect or bird. Briefly, a line is fit to this axis in each two-dimensional image (using *u*, *v* and *θ* from §2) and, together with the camera centre, is used to represent a plane in three-dimensional space. The best-fit line of intersection of the *n* planes is then found with a similar singular value decomposition algorithm ([44], §12.7).
